# Prevalence of Escherichia Coli in Urinary Tract Infection of Children Aged 1-15 Years in a Medical College of Eastern Nepal

**DOI:** 10.31729/jnma.4796

**Published:** 2020-01-31

**Authors:** Arun Giri, Raju Kafle, Ganesh Kumar Singh, Niraj Niraula

**Affiliations:** 1 Department of Pediatrics and Neonatology, Nobel Medical College Teaching Hospital, Biratnagar, Nepal; 2 Department of Pediatrics and Neonatology, Universal College of Medical Sciences, Bhairahawa, Nepal

**Keywords:** *antibiotic sensitivity*, *urinary tract infection*, *urine culture*

## Abstract

**Introduction:**

Urinary tract infection is one of the commonest causes of childhood morbidity. Early diagnosis and appropriate choice of antimicrobials is essential. Hence, this study aims to identify the prevalence of Escherichia coli in childhood urinary tract infections.

**Methods:**

This was a hospital based descriptive cross-sectional study conducted in Nobel Medical College, Biratnagar over a period of one year. A total of 163 cases aged 1-15 years were included and clinical profile, laboratory reports including bacterial isolates in urine cultures and their sensitivity patterns were documented.

**Results:**

The prevalence of Escherichia coli is 45 (53.57%) C.I. Escherichia coli was the most common organism isolated in bacterial cultures followed by Klebsiella 12 (14.29%), Enterococcus 10 (11.90%). Urinary tract infection was common among females with male: female ratio of 1:2.3. Fever 152 (93.2%) and abdominal pain 113 (69.3%) were the most common presenting symptoms. Escherichia coli was found most sensitive to Nitrofurantoin 43 (95.5%) followed by Ciprofloxacin 41 (91.1%) and Amikacin 40 (88.8%).

**Conclusions:**

Urinary tract infections in childhood require prompt attention and treatment to prevent significant morbidity and mortality. From this study it can be concluded that Escherichia coli is one of the most common isolates in urine culture and Aminoglycosides and Fluoroquinolones can be accepted as empirical treatment regimens for childhood Urinary tract infections.

## INTRODUCTION

Urinary tract infection (UTI) can be termed as a condition where one or several parts of the urinary system such as the ureter, urethra, kidney or bladder become infected. Approximately 95% of reported UTI cases are caused by bacteria that multiply at the entrance of the urethra and move up to the bladder. In some cases, the bacteria can ascend to the kidney and disseminate into the circulatory system.^1^

Paediatric UTI is associated with high morbidity and long term complications like renal scarring, hypertension, and chronic renal failure.^2^ In pediatric patients, UTI presents with nonspecific symptoms, making the diagnosis more confusing. Thus the high incidence of undiagnosed, improperly treated UTI in children is the cause for clinical and public concern. Urinary complaints are rare in children. The typical triad of abdominal/suprapubic pain, vomiting and fever with chills/rigors is the common presentation of upper and lower UTI generally appears only after 5 years of age.^3^

The main aim of this study is to find out the prevalence of E. coli in childhood urinary tract infections of a medical college.

## METHODS

This was a descriptive cross-sectional study conducted in Nobel medical college and teaching hospital, Biratnagar, Nepal over a period of one year from Nov 2018 to Dec 2019. After ethical clearance from institutional review committee, data was collected from Pediatric ward and OPD during the study period except those meeting the exclusion criteria.

Children 1 to 15 years of age who were suspected of having UTI were included in this study. Those who had received antibiotics or had undergone bladder catheterization within 48 hours prior to attending the hospital and specimens collected from techniques other than clean catch mid stream urine or samples which grew more than one type of organism were considered as contaminants and hence, excluded from this study.

$$\begin{array}{ll} \text{Sample size }(\text{n)} & \text{= }{\text{z}}^{2}\times \text{p q}/{\text{e}}^{\text{2}}\\ & \text{= }{(\text{1.64)}}^{\text{2}}\times \text{0.5}\times \text{(1}-\text{0.5)}/{\text{(0.1)}}^{\text{2}}\\ & \text{= }67 \end{array}$$

where,

Z = Confidence Interval, 90%
e = margin of error,10%
p = prevalence which was taken as 50%
q = (1-p)

Therefore, the calculated sample size was 67. Adding the 10% non-response rate, the sample size that will be taken would be 74. Convenience sampling method has been applied. The sample size has been doubled to 148 and 163 samples had been taken so as to increase the validity of the study.

After obtaining consent, children were asked to wash the perineum and the genitalia thoroughly with soap and water. In case of children below 5 years of age, the mother / guardian were instructed to do the same. The children and the mothers were asked to collect at least 10ml of the sample taking all the precautions to avoid contaminating the sample. A freshly voided clean catch mid stream urine sample was collected in a commercially obtained sterile, wide mouthed plastic container meant for urinalysis and urine culture.

Data collected was kept in Microsoft Excel and then edited and checked. After that the data was put in SPSS. Frequency, percentages was calculated for binary data and mean and standard deviation was calculated for continuous data after the normality of the data has been checked.

## RESULTS

The prevalence of E. coli is 45 (53.57%) C.I . Out of 163 cases included in the study, 48 (29.44%) were males and 115 (70.55%) were female with male: female ratio of 1:2.39. Highest number of cases were in the age group of 1-5 years 83 (50.9%).

Fever was the most common presenting symptom of urinary tract infection in children 152 (93.2%) followed by abdominal pain 113 (69.3%) and vomiting 78 (47.8%) (Table 1).

**Table 1:** Frequency of symptoms of UTI in children.

Symptoms	n (%)
Fever	152 (93.2)
Abdominal pain	113 (69.3)
Vomiting	78 (47.8)
Dysuria	63 (38.6)
Increased frequency of micturition	36 (22.2)
Constipation	24 (14.7)
Hematuria	20 (12.2)
Foul smelling urine	18 (11.04)

**Figure 1.**
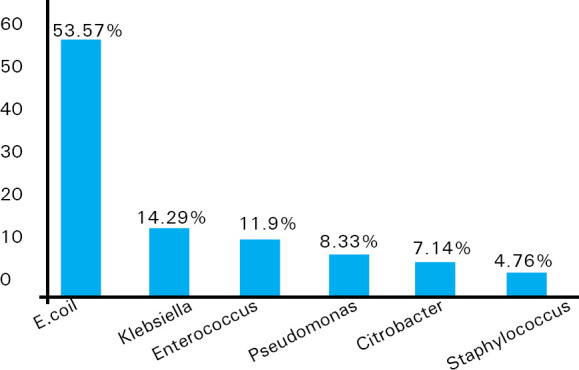
Bacterial isolates among positive cultures.

Eighty-four (52%) of the cases has culture positivity in urine culture reports. Commonest organism isolated in urine cultures was E.coli 45 (53.57) followed by Klebsiella pneumoniae 12 (14.29%), Enterococcus 10 (11.90%), Pseudomonas aeruginosa, Citrobacter and Staphylococcus aureus (Figure 1).

Among the bacterial isolates, E.coli was most sensitive to Nitrofurantoin 43 (95.5%), Ciprofloxacin 41 (91.1%) and Amikacin 40 (88.8%) and Klebsiella was most susceptible to Piperacillin-Tazobactum 7 (58.8%). Likewise, Enterococci and Pseudomonas were found to be most sensitive to Vancomycin and Piperacillin-Tazobactum respectively.

## DISCUSSION

This study was conducted to evaluate the etiological agents of urinary tract infections in children and their susceptibility to antimicrobial agents and provides laboratory data to allow comparison with other similar studies.

In this study, UTI was seen more frequently in girls (70.55%) than in boys (29.44%) which is consistent with the study conducted by Badhan R et al in India which showed female predominance of 54.2%.^4^ other studies conducted by Singh SD et al and Sharma A et al showed female preponderance of 67.4% and 65.0% respectively which are similar to our findings.^5,6^ The female predominance may be attributed to shorter female urethra and other additional factors.

Fever was the most common symptom of UTI in our study with 93.2% of the cases presenting with this symptom. Similar to our study, Singh SD et al and Shrestha LB et al found fever as the most common presenting symptom of UTI in children.^5,7^ Abdominal pain, vomiting and dysuria were other common presentations in our study which is shown by other studies as well in association with UTI in children.^8,9^

E. coli (53.57%) was the most common etiological agent isolated from urine cultures followed by Klebsiella (14.29%) in our study which is comparable to the study conducted by Kengne M et al which showed incidence of E.coli (59.3%) and Klebsiella (13.0%) respectively.^10^ Similarly, other studies conducted by Kaur N et al and Rezaee M et al showed predominance of E.coli followed by Klebsiella in their urine culture isolates.^7,8^

Antimicrobial sensitivity testing showed E.coli most sensitive to nitrofurantoin (95.5%) followed by ciprofloxacin (91.1%) and amikacin (88.8%) which was similar to the studies conducted by Sharma A et al.^6^ In contrast to our study, study conducted by Singh SD et al showed highest sensitivity to amikacin.^5^ Klebsiella showed highest sensitivity to ciprofloxacin(50.0%) in contrast to study conducted by Sharma et al which showed 100% sensitivity to ciprofloxacin.^6^ In our study ciprofloxacin was the most sensitive drug against all organisms.

## CONCLUSIONS

Infections of the urinary tract are one of the commonest infections in childhood and are a major threat for morbidity and mortality. So, larger studies at regular intervals must be carried out to determine the susceptibility patterns of the microbes to commonly used antimicrobials should be carried out. This study suggests use of ciprofloxacin, amikacin and nitofurantion for treatment of UTI in children wherever feasible based on antimicrobial susceptibility pattern in our region.

## Conflict of Interest

**None.**
